# Decreased thalamic monoamine availability in drug-induced parkinsonism

**DOI:** 10.1038/s41598-022-07773-5

**Published:** 2022-03-08

**Authors:** Yoon-Sang Oh, Sang-Won Yoo, Chul Hyoung Lyoo, Joong-Seok Kim

**Affiliations:** 1 Department of Neurology, College of Medicine, The Catholic University of Korea, Seoul, Republic of Korea; 2 Department of Neurology, Gangnam Severance Hospital, Yonsei University College of Medicine, Seoul, Republic of Korea; 3 Department of Neurology, Seoul St. Mary’s Hospital, College of Medicine, The Catholic University of Korea, 222, Banpo-daero, Seocho-gu, Seoul, 06591 Republic of Korea

**Keywords:** Movement disorders, Parkinson's disease

## Abstract

Drug-induced parkinsonism (DIP) is caused by a dopamine receptor blockade and is a major cause of misleading diagnosis of Parkinson’s disease (PD). Striatal dopamine activity has been investigated widely in DIP; however, most studies with dopamine transporter imaging have focused on the clinical characteristics and prognosis. This study investigated differences in striatal subregional monoamine availability among patients with DIP, normal controls, and patients with early PD. Thirty-five DIP patients, the same number of age-matched PD patients, and 46 healthy controls were selected for this study. Parkinsonian motor status was examined. Brain magnetic resonance imaging and positron emission tomography with ^18^F-N-(3-fluoropropyl)-2beta-carbon ethoxy-3beta-(4-iodophenyl) nortropane were performed, and the regional standardized uptake values were analyzed with a volume-of-interest template and compared among the groups. The groups were evenly matched for age, but there were numerically more females in the DIP group. Parkinsonian motor symptoms were similar in the DIP and PD groups. Monoamine availability in the thalamus of the DIP group was lower than that of the normal controls and similar to that of the PD group. In other subregions (putamen, globus pallidus, and ventral striatum), monoamine availability in the DIP group and normal controls did not differ and was higher than that in the PD group. This difference compared to healthy subject suggests that low monoamine availability in the thalamus could be an imaging biomarker of DIP.

## Introduction

Drug-induced parkinsonism (DIP) is the second most common cause of parkinsonism. The prevalence of DIP among individuals with parkinsonism varies from 15 to 40%^1,2^. The incidence rate of DIP is 3.3 to 13.9 per 100,000 person-years^2,3^. Dopamine receptor blocking agents such as prokinetics, antipsychotics, and antidepressants are the most common offending drugs^4^. Calcium channel blockers, antiarrhythmics, antiepileptics, and antihistamines also can cause DIP^4^.

The pathophysiological mechanism of DIP is regarded as a side effect of drugs that produce a D2 receptor blockade in the striatum^4^. Therefore, discontinuation of the offending drug resolves or improves parkinsonism. Dopamine transporter imaging is a useful tool for differentiating Parkinson’s disease (PD) from DIP and other tremor disorders^5^. In DIP patients, striatal dopamine uptake usually is normal, whereas PD patients show decreased dopamine activity, especially in the posterior putamen. Many studies have shown that dopamine transporter imaging has high sensitivity and specificity in differentiating PD from DIP^6^. However, few studies have examined whether striatal and extra-striatal monoamine transporter uptake in DIP is similar to that in normal healthy subjects.

We hypothesized that patients with DIP have different striatal monoamine availability patterns than normal controls. We investigated striatal subregional monoamine availability in patients with DIP and normal controls and analyzed differences in the striatal monoamine patterns of DIP patients, normal controls, and PD patients.

## Results

Totals of 35 patients with DIP, 35 age-matched patients with PD, and 46 healthy controls were enrolled in this study. The mean age was similar among the groups: DIP (69.8 ± 10.5 years), normal controls (69.3 ± 2.8 years), and PD (69.8 ± 11.0 years) (p = 0.953). Female sex was more predominant in the DIP group (80%) than in the PD (45.7%) and normal control (47.8%) groups (p = 0.004). The frequencies of hypertension, diabetes mellitus, and smoking did not differ among the three groups. The mean UPDRS total and part III scores in the DIP group were 20.0 ± 14.4, and 13.1 ± 8.1, respectively, which did not differ from the mean UPDRS total (23.6 ± 13.4, p = 0.283) and part III scores (14.0 ± 8.2, p = 0.642) in the PD group. The subscores of UPDRS part III including rest tremor (p = 0.120), rigidity (p = 0.584), and bradykinesia (p = 0.615) did not differ in DIP and PD group. The mean modified Hoehn and Yahr stage scores in the DIP group (2.0 ± 0.7) and PD group (1.8 ± 0.6) did not differ (p = 0.219) (Table 1).

**Table 1:** Clinical characteristics of the three groups.

	DIP (n = 35)	PD (n = 35)	Normal controls (n = 46)	P value	Post hoc test
Age, years, mean ± SD**	69.8 ± 10.5	69.8 ± 11.0	69.3 ± 2.8	0.953	DIP = NC = PD
Sex, male (%)*	7 (20.0)	19 (54.3)	24 (52.2)	0.004	DIP < NC = PD
Disease duration, years	1.2 ± 1.5	1.3 ± 2.3	–	0.781	–
Hypertension (%)*	11 (31.4)	14 (40.0)	22 (47.8)	0.329	DIP = NC = PD
Diabetes mellitus (%)*	12 (34.3)	8 (22.9)	11 (23.9)	0.478	DIP = NC = PD
Smoking status*				0.595	DIP = NC = PD
Non- or ex-smoker (%)	34 (97.1)	32 (91.4)	43 (93.5)		
Current smoker (%)	1 (2.9)	3 (8.6)	3 (6.5)		
UPDRS part III score	13.1 ± 8.1	14.0 ± 8.2	–	0.642	–
Rest tremor	2.2 ± 1.6	2.3 ± 2.3	–	0.120	
Rigidity	2.1 ± 2.0	1.7 ± 1.7	–	0.584	
Bradykinesia	6.1 ± 4.3	7.3 ± 4.1	–	0.615	
UPDRS total score	20.0 ± 14.4	23.6 ± 13.4	–	0.283	–
Hoehn and Yahr stage	2.0 ± 0.7	1.8 ± 0.6	–	0.219	–

Values represent mean with standard deviation or number of patients (percentage).

Analyses were performed using independent sample t-testing, *χ^2^ testing, and ** one way analysis of variance.

*UPDRS* Unified Parkinson’s Disease Rating Scale.

Among the drugs causing DIP, antipsychotics were the most common (n = 18, 51.4%), followed by prokinetics (n = 15, 42.9%) and antidepressants (n = 2, 5.7%). The most frequent offending drugs were levosulpiride (n = 12, 34.3%) and aripiprazole (n = 9, 25.7%) (Table 2). The parkinsonism of all DIP patients had resolved completely at 12 months after cessation of the offending drug.

**Table 2:** Offending drugs in patients with drug-induced parkinsonism.

	Number of patients (n = 35)
Gastro-prokinetics	15
Levosulpiride	12
Mosapride	3
Antipsychotics	18
Aripiprazole	9
Olanzapine	3
Risperidone	2
Amisulpride	1
Blonanserin	1
Quetiapine	1
Perphenazine	1
Antidepressants	2
Fluvoxamine	1
Duloxetine	1

**Figure 1:**
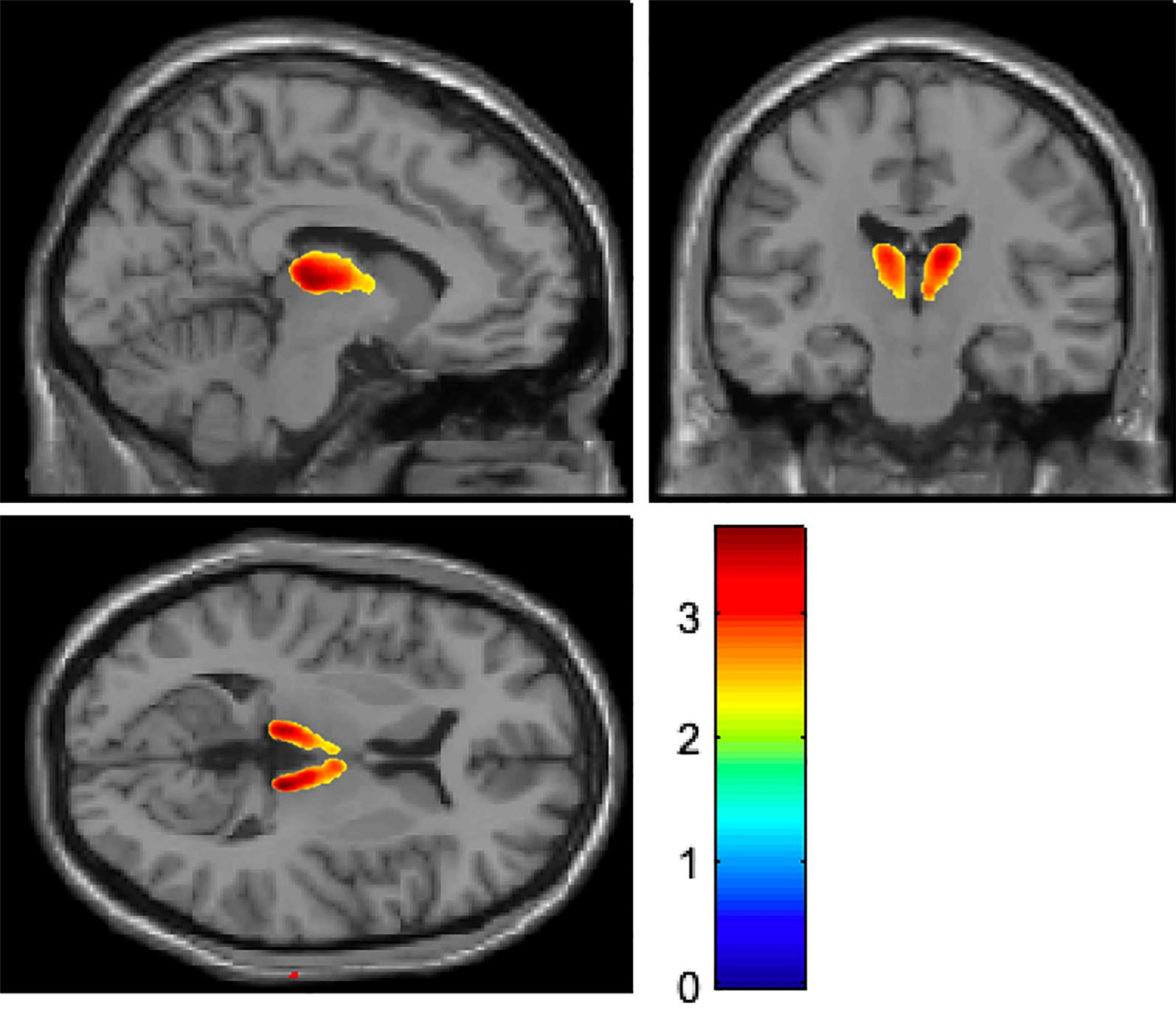
Voxel-based comparison of ^18^F-N-(3-fluoropropyl)-2beta-carbon ethoxy-3beta-(4-iodophenyl) nortropane positron emission tomography (^18^F-FP-CIT PET) between patients with drug-induced parkinsonism (DIP) and the healthy control group. Differences in standardized uptake value ratios (SUVRs) between the two groups (T values of Statistical Parametric Mapping) are visible in the colored region. High T value (red color) indicates that a large differences exists between two groups. The colored region showed a significant area of *p*-value set as 0.01. The SUVR values in the thalamus of patients with DIP were lower than those of healthy controls.

The SUVRs of the ^18^F-FP-CIT PET images were analyzed. The SUVRs of all subregions in the PD group were lower than those in the normal control group. The DIP group had SUVRs similar to those of the normal controls and higher than those of the PD group in the putamen, globus pallidus, and ventral striatum. The SUVR of the thalamus in the DIP group (1.43 ± 0.19) was significantly lower than that in the normal control group after adjusting age and sex (1.62 ± 0.13, p < 0.001) (Table 3, Fig. 1). These differences continued in the subgroup analysis when patients were further classified by sex (Supplementary Tables 1 and 2). UPDRS part III score and UPDRS total score were correlated with the SUVR values of putamen and globus pallidus in PD, whereas there were no correlations between UPDRS scores and striatal SUVR values in DIP (Supplementary Tables 3 and 4).

**Table 3:** Comparison of standardized uptake value ratios of the three groups.

Striatal subregion	DIP (n = 35)	PD (n = 35)	Normal controls (n = 46)	P value	Post hoc test
Caudate	4.69 ± 1.86 (1.81, 7.48)	3.92 ± 1.49 (1.81, 7.48)	4.85 ± 1.35 (2.32, 7.84)	0.019	NC > PD
Anterior caudate	4.95 ± 2.11 (1.45, 9.70)	4.13 ± 1.70 (1.75, 8.32)	5.15 ± 1.55 (2.27, 8.50)	0.023	NC > PD
Posterior caudate	3.54 ± 1.51 (0.93, 6.77)	2.93 ± 1.02 (1.30, 5.19)	3.86 ± 1.20 (1.39, 6.69)	0.002	NC > PD
Putamen	7.46 ± 1.38 (4.98, 10.86)	4.61 ± 1.66 (2.34, 9.39)	7.01 ± 1.27 (4.66, 10.30)	< 0.001	DIP = NC > PD
Anterior putamen	7.73 ± 1.60 (5.05, 11.33)	4.73 ± 1.83 (2.15, 10.11)	7.31 ± 1.49 (4.66, 10.85)	< 0.001	DIP = NC > PD
Posterior putamen	7.19 ± 1.47 (4.45, 10.29)	3.82 ± 1.84 (1.62, 9.01)	6.76 ± 1.40 (3.72, 9.85)	< 0.001	DIP = NC > PD
Ventral putamen	6.12 ± 1.43 (3.93, 10.85)	5.34 ± 1.42 (2.13, 6.74)	5.64 ± 0.93 (3.29, 8.14)	< 0.001	DIP = NC > PD
Globus pallidus	4.87 ± 1.00 (3.33, 7.71)	3.59 ± 1.19 (2.08, 7.72)	5.01 ± 1.04 (3.43, 7.54)	< 0.001	DIP = NC > PD
Thalamus	1.43 ± 0.19 (1.05, 1.81)	1.46 ± 0.15 (1.14, 1.81)	1.62 ± 0.13 (1.25, 1.93)	< 0.001	NC > DIP = PD
Ventral striatum	6.94 ± 1.40 (4.29, 10.45)	4.03 ± 1.08 (2.73, 9.18)	6.50 ± 1.53 (3.46, 9.58)	< 0.001	DIP = NC > PD

Values represent adjusted mean with standard deviation, and range.

Analyses were using analysis of covariance with Bonferroni post-hoc testing after controlling age and sex.

## Discussion

This study demonstrated that patients with DIP had a decreased monoamine uptake pattern in the thalamus compared with normal controls. Striatal monoamine availability in the other subregions of DIP patients was similar to that in normal controls. Patients with PD had decreased striatal monoamine availability compared with normal controls.

In this study, striatal monoamine availability was normal in DIP patients. Because parkinsonian symptoms in DIP patients are caused by blockade of post-synaptic dopamine receptors, dopamine transporter uptake should be normal in the striatum^5^. Although some DIP patients have reduced dopamine uptake, their subclinical parkinsonism was unmasked by anti-dopaminergic drugs and progressed after the offending drugs were stopped or their primary parkinsonism was worsened by the offending drug^4,5^. Our DIP patients had “pure” DIP, which means that their parkinsonian symptoms resolved after withdrawal of the offending drugs. Therefore, our study confirms that striatal monoamine availability in DIP is normal. However, our result differs from that of a recent study of dopamine transporters in symptomatic controls and healthy subjects that found symptomatic controls with essential tremor, vascular parkinsonism, or DIP to show higher putaminal dopamine transporter binding than healthy subjects^7^. Heterogeneity of symptomatic controls and selection bias in enrolling healthy subjects might explain that conflicting result.

In our study, only the SUVR of the thalamus differed significantly between the DIP group and normal controls. ^18^F-FP-CIT is a radioligand that binds not only to the striatum, but also to extra-striatal subregions such as the thalamus, hypothalamus, and brainstem^8,9^. The binding properties of the ^18^F-FP-CIT radioligand represent the availability of monoamine transporters such as dopamine, serotonin, and norepinephrine, and a decrease in its uptake implies impaired neural circuitry^8,9^. Serotonin is the main monoamine in the thalamus, and ^18^F-FP-CIT uptake in the thalamus also signifies serotonergic transmission from brainstem^8^. Our study supports a previous study showing that PD patients had reduced thalamic serotonin transporter binding compared with normal controls^9^ and reveals that DIP patients also have lower serotonin transporter binding in the thalamus than do normal controls.

The findings of this study are difficult to interpret. One possibility is that low thalamic serotoninergic availability is associated with increased risk of DIP. In PD, disruption of organized serotonergic control of the mesencephalic dopaminergic connections between the basal ganglia nuclei and across the basal ganglionic-cortico-thalamic circuits is involved in the onset of parkinsonian symptoms^10^. Therefore, the decreased monoamine uptake in the thalamus shown in this study could be associated with increased susceptibility to DIP upon exposure to an offending drug. Previous studies of the genetic polymorphism of serotonin receptors and transporters provide evidence of the development of DIP and extrapyramidal symptoms in a DIP patient and schizophrenic patients^11,12^. Additional explanation is that offending drugs such as selective serotonin reuptake inhibitors and many antipsychotics potentially block serotonin transporters in the thalamus^8^. Prokinetics act through the release of serotonin 5-hydroxytryptamine (5-HT), so the 5-HT_3_ antagonism and 5-HT_4_ agonism are the main mechanisms of prokinetics^13,14^. Mosapride^13^ and levosulpiride^14^ also act as 5-HT_3_ antagonists. As transmission to the central nervous system is mediated predominantly by a 5-HT_3_ antagonist^15^, serotonin uptake in the thalamus can be decreased by prokinetics. However, that hypothesis does not explain why these offending drugs do not affect presynaptic dopamine uptake in the striatum and affect the serotonin receptor only in the thalamus.

This study has several strengths and limitations. The major strength of our study is that we enrolled only patients with “pure” DIP whose symptoms resolved after withdrawal of the offending drug. DIP is a heterogeneous clinical syndrome; many patients have a full and long-lasting recovery with no subsequent PD, while other have persistent and worsening parkinsonian symptoms after discontinuation of the offending drug (subclinical parkinsonism or DIP unmasks PD) or recurrence of PD after complete remission from DIP (DIP antedates PD)^16^. The decreased striatal monoamine availability in DIP patients could be combined with subclinical PD and other atypical parkinsonism^4,5^. In addition, unlike most previous studies, we enrolled age-matched healthy subjects in our study. This study was conducted using a cross-sectional design and data from a PD registry, and we tried to match subject age using the similar patient matching method^17^. However, this study also has several limitations. First, we did not assess serotonergic binding uptake directly. Even though the ^18^F-FP-CIT radioligand has high serotonin affinity in the extra-striatal thalamus, serotonin transporter imaging might have shown better results. In addition, as we did not perform a follow-up PET scan with DIP patients, alteration of thalamic monoamine availability could not be confirmed. Second, we could not match the sex ratio among groups. A recent study conducted age and sex correlation of dopamine transporter imaging, which showed putaminal dopamine availability was about 10% higher in female than male^18^. However, sex effect was weaker than age effect, and sex effect was most prominent at young age^18^. In this study of subjects, most subjects were aged over 60 years old, and therefore we can suppose sex effect of striatal monoamine availability might be minimized. Moreover, we also reconfirmed our finding in the subgroup analyses classified by sex.

To our knowledge, few studies have investigated quantitative differences in striatal monoamine availability among DIP patients, normal controls, and PD patients. The DIP patients had striatal monoamine availability similar to that of the normal controls and essentially normal striatal monoamine availability compared with PD patients. On the other hand, monoamine availability in the thalamus of DIP patients was lower than that in the normal controls and similar to that in PD patients. Low thalamic monoamine uptake is characteristic of DIP patients compared to normal controls, although its clinical significance needs to be further investigated.

## Methods

This study protocol was approved by the Institutional Review Board at Seoul St. Mary’s Hospital, and all subjects provided written informed consent. All experiments were performed in accordance with relevant guidelines and regulations. The study is registered (Identification Number: KCT0005552 and KCT0006293) at the Clinical Research Information Service (CRIS; http://cris.nih.go.kr), an online clinical trial registration system established by the Korea Centers for Disease Control and Prevention (KCDC) with support from the Korea Ministry of Health and Welfare (KMOHW). This service is affiliated with the Primary Registries in the World Health Organization (WHO) Registry Network.

All experiments were performed in accordance with relevant guidelines and regulations.

### Subjects

Patients with diagnosed DIP who visited the movement disorder clinic in a university-affiliated hospital between January 2018 and December 2020 were enrolled. Age-matched PD patients newly diagnosed during the same period also were enrolled. DIP was defined using the following criteria: (1) presence of at least two of the four cardinal parkinsonian signs (tremor, rigidity, bradykinesia, and impaired postural reflexes); (2) absence of a personal history of extrapyramidal disorders before treatment with an offending drug; (3) onset of symptoms during the course of treatment with an offending drug; and (4) reversal of parkinsonian symptoms, although not necessarily completely, after discontinuation of the offending drug during follow-up of more than 6 months^19,20^. All DIP patients showed normal dopamine transporter uptake on a visual analysis of positron emission tomography (PET) with ^18^F-N-(3-fluoropropyl)-2beta-carbon ethoxy-3beta-(4-iodophenyl) nortropane (^18^F-FP-CIT). PD was diagnosed based on the UK Parkinson's Disease Society Brain Bank clinical diagnostic criteria^21^ and the Movement Disorder Society clinical diagnostic criteria for PD^22^. All PD patients had decreased dopamine transporter uptake in the striatum, mainly in the posterior putamen on a visual analysis. PD patients were enrolled from our research registry using the similar patient matching method^17^. Forty-six healthy subjects without any notable neurological or psychiatric diseases were recruited and included as controls. All subjects underwent brain magnetic resonance imaging (MRI) and did not demonstrate any abnormalities beyond mild white matter changes.

Demographics of age, sex, disease duration, medical history of hypertension and diabetes mellitus, and smoking status were collected. Parkinsonian motor symptoms were evaluated using the Unified Parkinson's Disease Rating Scale (UPDRS)^23^ and modified Hoehn and Yahr stage score^24^. All patients underwent brain MRI and ^18^F-FP-CIT PET at diagnosis. Normal subjects underwent brain MRI and ^18^F-FP-CIT PET with the same protocol as used for patients.

Patients were excluded using the following criteria: (1) atypical or other secondary parkinsonism, (2) previous stroke or structural lesions on the basal ganglia, or (3) use of anti-parkinsonian medications or medications that influence striatal monoamine uptake.

### PET imaging acquisition and processing

All ^18^F-FP-CIT PET and computed tomography (CT) images were acquired using the same PET/CT scanner. Patients received an intravenous injection (average 3.7 MBq/kg) of ^18^F-FP-CIT. Three hours later, brain CT scans were acquired for attenuation correction, followed by a 10-min ^18^F-FP-CIT emission PET scan. PET images were reconstructed in a 512 × 512 × 110 matrix using an ordered-subsets expectation maximization algorithm. The voxel size was 0.668 × 0.668 × 2 mm. Axial three-dimensional, T1-weighted, gradient-echo brain MRI (MP-RAGE) sequences (512 × 512 matrix, voxel spacing 0.469 × 0.469 × 1 mm) were acquired using a 3.0-T scanner (Magnetom Verio, Siemens, Erlangen, Germany).

Statistical Parametric Mapping 8 software (Wellcome Trust Centre for Neuroimaging, London, UK) implemented in MATLAB 2015a (MathWorks, Natick, MA, USA) was used for co-registration and spatial normalization of images and voxel-based comparisons. To spatially normalize the ^18^F-FP-CIT PET images, an MR-guided conventional spatial normalization method was used^25^. Then, PET images were co-registered to individual MR images and spatially normalized to the Montreal Neurological Institute space using parameter normalizing, skull-stripped MR^25^. Volume of interest (VOI) templates of striatal subregions were obtained after subcortical parcellation and partial volume correction using FreeSurfer 5.1 (Massachusetts General Hospital, Harvard Medical School; http://surfer.nmr.mgh.harvard.edu). VOI templates of four striatal subregions, the thalamus, and the cerebellum were normalized spatially to the MR template. Then subregional uptake values for each side of the caudate, putamen, globus pallidus, ventral striatum, thalamus, and cerebellum were calculated using the in-house MATLAB 2015a program (MathWorks, Natick, MA, USA) from our previous studies^26–28^. The mean standardized uptake value ratio (SUVR) was calculated as target SUV divided by cerebellar SUV.

### Statistical analysis

Statistical analyses were performed using SPSS software version 24.0 for Mac (IBM Corporation, New York, NY, USA). Pearson's χ^2^ test was used to compare the frequencies of categorical variables. Independent sample *t*-testing was used to compare means between the DIP and PD groups, and one-way analysis of variance testing was used to compare means among the three groups. The subregional monoamine uptake of the three groups was controlled for age and sex and analyzed using one-way analysis of covariance with Bonferroni post-hoc testing. Spearman’s correlations between UPDRS scores and the SUVR values of striatum in PD and DIP were analyzed. Statistical significance was set at p < 0.05.

## Supplementary Information

Supplementary Information.

## Data Availability

Anonymized data generated during the current study are available from the corresponding author on reasonable request from individuals affiliated with research or health care institutions.
