# Author Correction: Decreased thalamic monoamine availability in drug-induced parkinsonism

**DOI:** 10.1038/s41598-022-20205-8

**Published:** 2022-09-14

**Authors:** Yoon-Sang Oh, Sang-Won Yoo, Chul Hyoung Lyoo, Joong-Seok Kim

**Affiliations:** 1 Department of Neurology, College of Medicine, The Catholic University of Korea, Seoul, Republic of Korea; 2 Department of Neurology, Gangnam Severance Hospital, Yonsei University College of Medicine, Seoul, Republic of Korea; 3 Department of Neurology, Seoul St. Mary’s Hospital, College of Medicine, The Catholic University of Korea, 222, Banpo-daero, Seocho-gu, Seoul, 06591 Republic of Korea

Correction to: *Scientific Reports* 10.1038/s41598-022-07773-5, published online 08 March 2022

The original version of this Article omitted an affiliation for Yoon‑Sang Oh and Sang‑Won Yoo. Their correct affiliation is listed below:

Department of Neurology, College of Medicine, The Catholic University of Korea, Seoul, Republic of Korea

The original Article has been corrected.

