# Supplementary material for: Decreased thalamic monoamine availability in drug-induced parkinsonism
Source: Sci Rep. 2022 Mar 8;12:3749. doi: 10.1038/s41598-022-07773-5 (PMC8904448; doi:10.1038/s41598-022-07773-5)
Supplement: Supplementary file 1 — Supplementary Information. [file 41598_2022_7773_MOESM1_ESM.pdf]

Supplementary Table 1. Comparison of standardized uptake value ratios of the three groups in men

Striatal subregion	DIP (n = 7)	PD (n = 19)	Normal controls (n = 24)	P value	Post hoc test
Caudate	3.84 (1.20)	3.84 (1.59)	4.58 (1.23)	0.116	NC = DIP = PD
Anterior caudate	3.99 (1.37)	4.02 (1.83)	4.82 (1.40)	0.127	NC = DIP = PD
Posterior caudate	2.80 (1.20)	2.94 (1.11)	3.47 (1.06)	0.146	NC = DIP = PD
Putamen	6.69 (1.04)	4.72 (1.45)	6.92 (1.08)	< 0.001	DIP = NC > PD
Anterior putamen	6.94 (1.11)	4.86 (1.62)	7.14 (1.17)	< 0.001	DIP = NC > PD
Posterior putamen	6.29 (1.25)	3.89 (1.68)	6.60 (1.17)	< 0.001	DIP = NC > PD
Ventral putamen	5.34 (1.01)	4.00 (0.94)	5.59 (0.91)	< 0.001	DIP = NC > PD
Globus pallidus	4.85 (0.39)	3.76 (1.27)	4.76 (0.79)	0.004	NC > PD, NC= DIP
Thalamus	1.40 (0.16)	1.51 (0.14)	1.61 (0.13)	0.002	NC > DIP = PD
Ventral striatum	5.34 (1.01)	4.00 (0.94)	5.59 (0.91)	< 0.001	DIP = NC > PD

Values represent adjusted mean with standard deviation.
Analyses were controlled for age and performed using analysis of covariance with Bonferroni post-hoc testing.

Supplementary Table 2. Comparison of standardized uptake value ratios of the three groups in women

Striatal subregion	DIP (n = 28)	PD (n = 16)	Normal controls (n = 22)	P value	Post hoc test
Caudate	4.91 (1.95)	4.00 (1.40)	5.14 (1.44)	0.175	NC = DIP = PD
Anterior caudate	5.20 (2.21)	4.25 (1.60)	5.52 (1.67)	0.206	NC = DIP = PD
Posterior caudate	3.72 (1.54)	2.91 (0.94)	4.28 (1.23)	0.013	NC > PD, NC= DIP
Putamen	7.65 (1.40)	4.48 (1.93)	7.10 (1.48)	< 0.001	DIP = NC > PD
Anterior putamen	7.93 (1.66)	4.58 (2.10)	7.51 (1.79)	< 0.001	DIP = NC > PD
Posterior putamen	7.41 (1.45)	3.74 (2.07)	6.95 (1.61)	< 0.001	DIP = NC > PD
Ventral putamen	6.31 (1.47)	4.05 (1.26)	5.68 (0.97)	< 0.001	DIP = NC > PD
Globus pallidus	4.87 (1.11)	3.38 (1.09)	5.28 (1.22)	< 0.001	DIP = NC > PD
Thalamus	1.44 (0.15)	1.40 (0.15)	1.63 (0.13)	< 0.001	NC > DIP = PD
Ventral striatum	7.09 (1.49)	5.23 (1.70)	6.35 (1.79)	0.004	DIP > PD

Values represent adjusted mean with standard deviation.
Analyses were controlled for age and performed using analysis of covariance with Bonferroni post-hoc testing.

Supplementary Table 3. Spearman's correlation between UPDRS scores and the standardized uptake value ratio (SUVR) values of striatum in Parkinson's disease

Striatal subregion	UPDRS Part I	UPDRS Part II	UPDRS Part III	Tremor subscore	Rigidity subscore	Bradykinesia subscore	UPDRS Total
Caudate	-0.303 (0.077)	-0.129 (0.461)	0.024 (0.889)	0.003 (0.987)	-0.057 (0.749)	-0.018 (0.916)	-0.014 (0.553)
Putamen	-0.149 (0.393)	-0.301 (0.079)	-0.364 (0.031)*	-0.299 (0.081)	-0.358 (0.035)*	0.309 (0.071)	-0.385 (0.022)*
Globus pallidus	0.027 (0.880)	-0.324 (0.058)	-0.485 (0.003)**	-0.405 (0.016)*	-0.426 (0.011)*	-0.385 (0.023)*	-0.443 (0.008)**
Thalamus	-0.310 (0.070)	-0.200 (0.248)	-0.006 (0.971)	0.150 (0.389)	-0.056 (0.748)	-0.097 (0.581)	-0.171 (0.326)
Ventral striatum	-0.338 (0.047)*	-0.401 (0.017)*	-0.303 (0.077)	-0.208 (0.230)	-0.274 (0.112)	-0.277 (0.107)	-0.416 (0.013)

Data are r (p-value).
* $p < 0.05$, ** $p < 0.01$

Supplementary Table 4. Spearman's correlation between UPDRS scores and the standardized uptake value ratio (SUVR) values of striatum in drug-induced Parkinsonism

Striatal subregion	UPDRS Part I	UPDRS Part II	UPDRS Part III	Tremor subscore	Rigidity subscore	Bradykinesia subscore	UPDRS Total
Caudate	0.151 (0.387)	-0.073 (0.676)	-0.223 (0.198)	-0.122 (0.486)	0.152 (0.383)	-0.138 (0.429)	-0.172 (0.324)
Putamen	0.237 (0.170)	0.071 (0.685)	-0.157 (0.369)	-0.101 (0.565)	0.165 (0.343)	0.063 (0.721)	-0.090 (0.609)
Globus pallidus	-0.059 (0.738)	0.000 (0.999)	0.023 (0.895)	-0.092 (0.599)	-0.155 (0.373)	0.100 (0.566)	-0.012 (0.945)
Thalamus	0.186 (0.284)	-0.007 (0.969)	-0.064 (0.714)	-0.158 (0.364)	-0.048 (0.786)	-0.137 (0.432)	-0.042 (0.813)
Ventral striatum	0.097 (0.578)	-0.069 (0.693)	-0.243 (0.159)	-0.220 (0.204)	0.090 (0.606)	-0.084 (0.630)	-0.202 (0.245)

Data are r (p-value).
